# 依托泊苷联合G-CSF与环磷酰胺联合G-CSF方案在新诊断多发性骨髓瘤患者自体外周造血干细胞动员中的比较

**DOI:** 10.3760/cma.j.issn.0253-2727.2022.09.012

**Published:** 2022-09

**Authors:** 菁 李, 闰 张, 丽娟 陈, 晓燕 屈, 化 陆, 建勇 李, 媛媛 金

**Affiliations:** 江苏省人民医院，南京医科大学第一附属医院血液科，南京 210029 Department of Hematology, the First Affiliated Hospital of Nanjing Medical University, Jiangsu Province Hospital, Nanjing 210029, China

自体造血干细胞移植（auto-HSCT）可延长多发性骨髓瘤（MM）患者无进展生存期并降低微小残留病（MRD）水平[1]。GRIFFIN和MASTER临床试验均将auto-HSCT作为新诊断MM（NDMM）患者的一线治疗选择[2]–[3]。因此，auto-HSCT仍是部分MM患者的早期治疗选择[4]。auto-HSCT成功的首要条件是动员获得足够数量的CD34^+^造血干细胞，此外还需要考虑方案的经济效益以及动员相关不良反应。《中国多发性骨髓瘤诊治指南（2020年修订）》推荐大剂量环磷酰胺联合粒细胞集落刺激因子（G-CSF）或CXCR4拮抗剂作为MM患者自体外周造血干细胞动员方案[5]，依托泊苷联合G-CSF在血液系统恶性肿瘤患者自体外周造血干细胞动员中也有良好的效果[6]–[7]。我们对近年来在本中心接受依托泊苷联合G-CSF和环磷酰胺联合G-CSF两种方案进行自体外周造血干细胞动员的NDMM患者进行回顾性分析，比较两种动员方案的效果和安全性。

## 病例与方法

1. 病例：纳入2018年1月至2021年4月期间在江苏省人民医院进行自体外周造血干细胞动员的NDMM患者84例。所有患者均符合2016年国际骨髓瘤工作组（IMWG）诊断标准[8]且经诱导治疗获得部分缓解（PR）及以上疗效。其中54例患者采用依托泊苷联合G-CSF动员方案（依托泊苷+G-CSF组），30例采用环磷酰胺联合G-CSF动员方案（环磷酰胺+G-CSF组）。本研究获得南京医科大学第一附属医院江苏省人民医院伦理委员会批准（2022-SR-448）。

2. 造血干细胞动员和采集：依托泊苷+G-CSF组：单剂依托泊苷1.6 g/m^2^原液静脉泵入（10 h）；环磷酰胺+G-CSF组：环磷酰胺4 g/m^2^分2 d静脉滴注；两组患者均从动员第5天直至干细胞采集当天予以G-CSF10 µg/kg皮下注射。当患者白细胞计数恢复到>4×10^9^/L时，采用COBE Spectra（Terumo BCT公司）血细胞分离机进行单个核细胞采集，采用流式细胞术进行CD34^+^细胞计数。采集成功：CD34^+^细胞≥2.0×10^6^/kg；采集失败：CD34^+^细胞<2.0×10^6^/kg；理想采集：CD34^+^细胞≥5.0×10^6^/kg。

3. 移植预处理及造血干细胞回输：预处理方案为美法仑200 mg/m^2^或140 mg/m^2^分2 d静脉滴注（移植前2 d、1 d），回输造血干细胞（CD34^+^细胞>2×10^6^/kg）后第1天开始使用G-CSF 10 µg·kg^−1^·d^−1^，直至外周血中性粒细胞绝对计数（ANC）恢复到1.0×10^9^/L。

4. 随访和疗效评价：粒细胞植入定义为中性粒细胞绝对计数（ANC）≥0.5×10^9^/L连续3 d，血小板植入定义为PLT≥20×10^9^/L连续7 d且脱离血小板输注。采用美国卫生及公共服务部常见不良事件评价标准（CTCAE 5.0）评价动员相关不良反应。移植后100 d内进行第1次住院随访，维持治疗阶段每3个月进行1次门诊随访，随访截止时间为2021年7月。参照IMWG标准[8]进行疗效评价。

5. 统计学处理：应用SPSS 26.0软件进行统计学分析。分类变量以数字（百分比）来表示并采用*χ*^2^检验，矫正*χ*^2^或Fisher检验评估。符合正态分布数据用“均数±标准差”表示，不符合正态分布数据用“中位数（最小值～最大值）”表示并采用Mann-Whitney *U*检验进行组间比较。*P*<0.05为差异有统计学意义。

## 结果

1. 临床特征：84例接受auto-HSCT的NDMM患者，依托泊苷+G-CSF组54例，环磷酰胺+G-CSF组30例。两组除中位年龄差异具有统计学意义（*P*＝0.015）外，其他临床指标差异均无统计学意义（*P*>0.05），详见表1。

**表1:** 84例行自体造血干细胞动员多发性骨髓瘤患者的临床特征

指标	环磷酰胺+G-CSF组（30例）	依托泊苷+G-CSF组（54例）	统计量	*P*值
中位年龄［岁，*M*（范围）］	54（27~64）	56（30~68）	*z*=−2.432	0.015
性别［例（男/女）］	13/17	28/26	*χ*^2^=0.560	0.454
ISS分期［例（％）］			*χ*^2^=3.704	0.157
Ⅰ期	6（25.0）	10（18.5）		
Ⅱ期	14（58.3）	20（37.0）		
Ⅲ期	4（16.7）	24（44.5）		
骨髓瘤分型［例（％）］				0.499
IgG型	12（40.0）	27（50.0）		
IgA型	11（36.6）	15（27.8）		
IgD型	2（6.7）	1（1.9）		
轻链型	5（16.7）	11（20.3）		
诱导方案［例（％）］				0.425
基于硼替佐米方案	14（46.7）	20（37.0）		
硼替佐米+来那度胺+地塞米松	13（43.3）	31（57.4）		
传统化疗	3（10.0）	3（5.6）		
治疗线数［线，*M*（范围）］	1（1~3）	1（1~3）	*z*=−0.387	0.698
诱导治疗周期［个，*M*（范围）］	4（3~8）	4（3~9）	*z*=−0.294	0.769
来那度胺治疗周期［个，*M*（范围）］	2.5（0~6）	3（0~5）	*z*=−0.231	0.817
动员前疾病状态［例（％）］				0.872
CR	17（56.7）	29（53.7）		
VGPR	12（40.0）	21（38.9）		
PR	1（3.3）	4（7.4）		

注：G-CSF：粒细胞集落刺激因子；CR：完全缓解；VGPR：非常好的部分缓解；PR：部分缓解

2. 自体造血干细胞采集效果比较：环磷酰胺+G-CSF组采集成功率低于依托泊苷+G-CSF组［76.7％（23/30）对98.1％（53/54），*P*＝0.005］。采集成功患者中，依托泊苷+G-CSF组中位采集次数少于环磷酰胺+G-CSF组（*P*＝0.003），且依托泊苷+G-CSF组单次采集成功的患者比例高于环磷酰胺+G-CSF组（*P*＝0.018）。依托泊苷+G-CSF组获得理想采集、首次采集获得理想采集的患者比例均高于环磷酰胺+G-CSF组（*P*＝0.009，*P*＝0.001）。依托泊苷+G-CSF组动员至首次采集中位时间短于环磷酰胺+G-CSF组（*P*<0.001）。在采集成功的患者中，依托泊苷+G-CSF组CD34^+^细胞总采集量高于环磷酰胺+G-CSF组（*P*＝0.001），第1天CD34^+^细胞采集量也高于环磷酰胺+G-CSF组（*P*＝0.001），第2、3天CD34^+^细胞采集量差异无统计学意义。详见表2。

**表2:** 2种自体外周造血干细胞动员方案在新诊断多发性骨髓瘤患者动员结果的比较

指标	环磷酰胺+G-CSF组（30例）	依托泊苷+G-CSF组（54例）	统计量	*P*值
采集成功［例（％）］	23（76.7）	53（98.1）	*χ*^2^=7.986	0.005
中位采集次数［次，*M*（范围）］	2（1~3）	1（1~3）	*z*=−2.971	0.003
单次采集成功［例（％）］	18（60.0）	45（83.3）	*χ*^2^=5.600	0.018
获得理想采集量［例（％）］	13（43.3）	39（72.2）	*χ*^2^=6.825	0.009
单次采集获得理想采集量［例（％）］	8（26.7）	35（64.8）	*χ*^2^=11.233	0.001
动员至首次采集中位时间［d，*M*（范围）］	12（10~13）	10（9~13）	*z*=−5.223	<0.001
CD34^+^细胞总采集量［×10^6^/kg，*M*（范围）］	5.02（2.01~13.8）	11.17（2.15~30.80）	*z*=−3.358	0.001
第1天CD34^+^细胞采集量［×10^6^/kg，*M*（范围）］	3.32（0.36~13.83）	10.68（0.31~30.74）	*z*=−3.386	0.001
第2天CD34^+^细胞采集量［×10^6^/kg，*M*（范围）］	1.59（0.58~8.32）	2.73（1.21~6.51）	*z*=−1.231	0.238
第3天CD34^+^细胞采集量［×10^6^/kg，*M*（范围）］	0.84（0.73~0.95）	1.12（0.96~1.29）	*z*=−1.549	0.121

注：采集失败：CD34^+^细胞总采集量<2×10^6^/kg；采集成功：CD34^+^细胞总采集量≥2×10^6^/kg；理想的采集：CD34^+^细胞总采集量≥5×10^6^/kg

3. 动员相关不良反应：两组患者非血液学不良反应（黏膜炎、肺炎和血流感染）发生率差异无统计学意义。最常见的不良反应为中性粒细胞减少伴发热，环磷酰胺+G-CSF组、依托泊苷+G-CSF组分别有11例（36.7％）、20例（37.0％），两组发生率差异无统计学意义（*P*＝0.973）；依托泊苷+G-CSF组需要血小板输注（PLT<20×10^9^/L时）的患者比例高于环磷酰胺+G-CSF组（14.8％对0％，*P*＝0.067）。详见表3。

**表3:** 两种多发性骨髓瘤自体造血干细胞动员方案的不良反应比较［例（％）］

组别	例数	粒细胞减少伴发热	肺炎	血流感染	黏膜炎	血小板输注
环磷酰胺+G-CSF组	30	11（36.7）	4（13.3）	1（3.3）	1（3.3）	0（0）
依托泊苷+G-CSF组	54	20（37.0）	3（5.6）	0（0）	2（3.7）	8（14.8）

*χ*^2^值		0.001	0.679	1.822	<0.001	3.343
*P*值		0.973	0.410	0.177	1.000	0.067

4. 干细胞动员效率的预测因素：对影响干细胞采集成功的因素进行分析，纳入年龄、性别、疾病分期、诱导治疗方案、诱导治疗疗程数、来那度胺使用周期、动员时疾病状态、动员方案以及疾病诊断到动员时间，结果发现以上诱导治疗中来那度胺使用周期数和动员方案是影响干细胞采集成功的独立因素，来那度胺使用次数增多是采集成功的不良因素［*OR*＝0.431，95％*CI*（0.187～0.994），*P*＝0.048］，依托泊苷是采集成功的独立良好因素［*OR*＝16.386，95％*CI*（1.640～163.785），*P*＝0.017］。进一步分析影响理想采集的因素，来那度胺诱导治疗疗程是获得理想采集的不良因素［*OR*＝0.711，95％*CI*（0.545～0.926），*P*＝0.012］，依托泊苷动员是获得采集理想的独立良好因素［*OR*＝4.221，95％*CI*（1.522～11.705），*P*＝0.006］。

5. auto-HSCT后造血重建：除1例患者因间质性肺炎延迟进行auto-HSCT外，其他动员成功的患者均行一线auto-HSCT，其中环磷酰胺+G-CSF组23例（76.7％），依托泊苷+G-CSF组52例（96.3％）。2例患者因有高危遗传学因素，计划进行二次auto-HSCT，因此在第一次auto-HSCT中回输了半数采集量的CD34^+^细胞。在接受大剂量美法仑预处理后，依托泊苷+G-CSF组回输中位CD34^+^细胞数为10.98（2.15～30.80）×10^6^/kg，环磷酰胺+G-CSF组为5.02（2.01～13.8）×10^6^/kg（*P*＝0.001）。依托泊苷+G-CSF组、环磷酰胺+G-CSF组的中性粒细胞植入中位时间分别为9（8～14）d、10（8～14）d（*P*＝0.007），血小板植入的中位时间分别为9（7～15）d、10（5～14）d（*P*＝0.059）。

## 讨论

根据美国血液和骨髓移植协会建议，自体造血干细胞动员的最低采集目标是CD34^+^细胞≥2×10^6^/kg，但更多CD34^+^干细胞回输有助于确保患者移植后造血重建的快速建立，同时最好能采集到两次移植需要的干细胞，以用于二次移植或晚期移植[9]–[10]。目前，环磷酰胺（3～4 g/m^2^）+G-CSF是MM患者最常用的自体造血干细胞动员方案[11]–[12]。多个研究显示，依托泊苷在自体造血干细胞动员中也有较好的效果，文献中报道的依托泊苷的剂量由375 mg/m^2^至2.4 g/m^2^不等[7],[13]–[15]。在本组病例中，我们选择了1.6 g/m^2^这一被认为有效且安全的剂量[15]–[16]，发现与传统的环磷酰胺4 g/m^2^方案相比，该方案可获得更高的采集成功率（98.1％）和理想采集率（72.2％）。更重要的是依托泊苷动员组单次理想采集较率高，极大地节约了经济成本。自体造血干细胞动员失败的危险因素多种多样，包括高龄、既往放疗或广泛化疗、既往来那度胺或嘌呤类似物治疗、既往动员失败[10]。本研究未发现影响采集成功率的危险因素，但发现环磷酰胺动员方案是取得理想采集量的独立危险因素（*HR*＝6.222）。

化疗联合G-CSF动员的局限性主要是安全性问题，本研究中依托泊苷+G-CSF组与环磷酰胺+G-CSF组不良反应相似。其中，中性粒细胞减少伴发热是最常见的3级以上不良反应，在依托泊苷+G-CSF组与环磷酰胺+G-CSF组发生率相似（37.0％对36.7％，*P*＝0.973）。这些患者在给予抗生素1～2 d后，发热均能得到控制。在血液学不良反应中，依托泊苷+G-CSF组因PLT<20×10^9^/L输注血小板事件增多，但差异无统计学意义（*P*＝0.067），且患者在此动员过程中仅需要1次血小板输注。而在后续造血干细胞回输后，依托泊苷+G-CSF组中性粒细胞和血小板恢复较环磷酰胺+G-CSF组迅速。这一优势有助于降低感染风险和治疗费用。

CXCR4拮抗剂是一种新型非化疗动员剂，但昂贵的价格使其临床应用受限。化疗药物被用于自体造血干细胞动员最初是为了减轻肿瘤负荷并提高患者的缓解深度。环磷酰胺被认为是一种对骨髓瘤细胞有显著抑制活性且对疾病控制有益的药物[17]。Uy等[18]对968例接受auto-HSCT的MM患者进行分析，显示动员方法的选择不影响auto-HSCT后总生存时间（OS）以及无疾病进展时间（PFS），提示化疗动员在新药时代对疾病的控制并无明显助益。因此，化疗药物的效果和不良反应可能是动员剂选择的主要参考因素。本研究发现，依托泊苷化疗动员较环磷酰胺化疗动员在采集次数上更具优势，不良反应反应可控，但需要血小板输注的患者比例较高，后续应探索优化依托泊苷剂量，在获得较好的动员效果的同时降低血液学毒性，进一步提高安全性。

综上所述，本研究结果显示依托泊苷联合G-CSF较环磷酰胺联合G-CSF动员方案更为有效，不良反应可控。上述结论尚需进行大样本临床研究加以验证并延长随访时间观察对长期生存的影响。

## References

[1] Attal M, Lauwers-Cances V, Hulin C (2017). Lenalidomide, Bortezomib, and Dexamethasone with Transplantation for Myeloma[J]. N Engl J Med.

[2] Voorhees PM, Kaufman JL, Laubach J (2020). Daratumumab, lenalidomide, bortezomib, and dexamethasone for transplant-eligible newly diagnosed multiple myeloma: the GRIFFIN trial[J]. Blood.

[3] Costa LJ, Chhabra S, Godby KN (2019). Daratumumab, Carfilzomib, Lenalidomide and Dexamethasone (Dara-KRd) induction, autologous transplantation and post-transplant, response-adapted, measurable residual disease (MRD)-based Dara-Krd consolidation in patients with newly diagnosed multiple myeloma (NDMM)[J]. Blood.

[4] Fu S, Wu CF, Wang M (2019). Cost effectiveness of transplant, conventional chemotherapy, and novel agents in multiple myeloma: a systematic review[J]. Pharmacoeconomics.

[5] 中国医师协会血液科医师分会, 中华医学会血液学分会, 中国医师协会多发性骨髓瘤专业委员会 (2020). 中国多发性骨髓瘤诊治指南(2020年修订)[J]. 中华内科杂志.

[6] Junghanss C, Leithäuser M, Wilhelm S (2001). High-dose etoposide phosphate and G-CSF mobilizes peripheral blood stem cells in patients that previously failed to mobilize[J]. Ann Hematol.

[7] Gianni AM, Bregni M, Siena S (1992). Granulocyte-macrophage colony-stimulating factor or granulocyte colony-stimulating factor infusion makes high-dose etoposide a safe outpatient regimen that is effective in lymphoma and myeloma patients[J]. J Clin Oncol.

[8] Kumar S, Paiva B, Anderson KC (2016). International Myeloma Working Group consensus criteria for response and minimal residual disease assessment in multiple myeloma[J]. Lancet Oncol.

[9] Stiff PJ, Micallef I, Nademanee AP (2011). Transplanted CD34(+) cell dose is associated with long-term platelet count recovery following autologous peripheral blood stem cell transplant in patients with non-Hodgkin lymphoma or multiple myeloma[J]. Biol Blood Marrow Transplant.

[10] Giralt S, Costa L, Schriber J (2014). Optimizing autologous stem cell mobilization strategies to improve patient outcomes: consensus guidelines and recommendations[J]. Biol Blood Marrow Transplant.

[11] Hamadani M, Kochuparambil ST, Osman S (2012). Intermediate-dose versus low-dose cyclophosphamide and granulocyte colony-stimulating factor for peripheral blood stem cell mobilization in patients with multiple myeloma treated with novel induction therapies[J]. Biol Blood Marrow Transplant.

[12] Hiwase DK, Bollard G, Hiwase S (2007). Intermediate-dose CY and G-CSF more efficiently mobilize adequate numbers of PBSC for tandem autologous PBSC transplantation compared with low-dose CY in patients with multiple myeloma[J]. Cytotherapy.

[13] Park Y, Kim DS, Jeon MJ (2019). Single-dose etoposide is an effective and safe protocol for stem cell mobilization in patients with multiple myeloma[J]. J Clin Apher.

[14] Mahindra A, Bolwell BJ, Rybicki L (2012). Etoposide plus G-CSF priming compared with G-CSF alone in patients with lymphoma improves mobilization without an increased risk of secondary myelodysplasia and leukemia[J]. Bone Marrow Transplant.

[15] Kanfer E, McGuigan D, Samson D (1998). High-dose etoposide with granulocyte colony-stimulating factor for mobilization of peripheral blood progenitor cells: efficacy and toxicity at three dose levels[J]. Br J Cancer.

[16] Junghanss C, Leithauser M, Wilhelm S (2001). High-dose etoposide phosphate and G-CSF mobilizes peripheral blood stem cells in patients that previously failed to mobilize[J]. Ann Hematol.

[17] Dingli D, Nowakowski GS, Dispenzieri A (2006). Cyclophosphamide mobilization does not improve outcome in patients receiving stem cell transplantation for multiple myeloma[J]. Clin Lymphoma Myeloma.

[18] Uy GL, Costa LJ, Hari PN (2015). Contribution of chemotherapy mobilization to disease control in multiple myeloma treated with autologous hematopoietic cell transplantation[J]. Bone Marrow Transplant.

